# Diagnosis of Distal Cholangiocarcinoma after the Removal of Choledocholithiasis

**DOI:** 10.1155/2012/396869

**Published:** 2012-11-22

**Authors:** Yasuhiro Ito, Takeshi Kenmochi, Tomohisa Egawa, Shinobu Hayashi, Atsushi Nagashima, Yuko Kitagawa

**Affiliations:** ^1^Department of Surgery, Saiseikai Yokohamashi Tobu Hospital, 3-6-1 Shimosueyoshi, Tsurumi-ku, Yokohama-shi, Kanagawa 230-8765, Japan; ^2^Department of Surgery, Keio University School of Medicine, 35 Shinanomachi, Shinjuku-ku, Tokyo 160-8582, Japan

## Abstract

*Background and Aim*. Distal cholangiocarcinoma associated with choledocholithiasis has not been reported, and the causal relationship remains to be established. We evaluated diagnosis of distal cholangiocarcinoma diagnosed after the removal of choledocholithiasis. *Patients and Methods*. We assigned 9 cases of cholangiocarcinoma with choledocholithiasis to Group A. As a control group, 37 patients with cholangiocarcinoma without choledocholithiasis were assigned to Group B. *Results*. Abdominal pain at admission is the only significant difference between Group A and Group B (*P* = 0.001). All patients in Group A had gall bladder stones, compared with 7 patients (19%) in Group B (*P* < 0.01). Of the 9 patients in Group A, endoscopic retrade cholangiopancreatography (ERCP) detected normality in 2 patients (22%) and abnormalities in 7 patients (78%). Of the 32 patients in Group B, ERCP detected normality in 4 patients (13%) and abnormalities in 28 patients (88%) (*P* = 0.597). Intraductal ultrasonography (IDUS) detected a tumor in 8 patients in Group A, while in Group B, IDUS detected normality in 1 patient (3%) and tumors in 29 patients (97%) (*P* = 1.000). *Conclusions*. IDUS after stone removal may potentially help in the detection of unexpected tumors. Therefore, we believe that IDUS after stone removal will lead to improve outcome and prognosis.

## 1. Introduction

The frequency of cholangiocarcinoma is increasing globally, and it currently accounts for 3% of all gastrointestinal cancers [1]. The 5-year survival rates of patients with perihilar and distal tumors have been reported to be 10% and 23%, respectively [2]. Early cholangiocarcinoma is difficult to diagnose because the symptoms usually occur late in the disease. Because these tumors tend to invade the surrounding vessels and nerves, most patients have unresectable disease at diagnosis and poor survival. The prognosis remains unsatisfactory even if the patient undergoes extensive surgery, which is the only curative treatment for these tumors. Therefore, early detection and diagnosis are needed to improve long-term survival. Reports of distal cholangiocarcinoma associated with choledocholithiasis are very rare, and the causal relationship is not established despite the fact that intrahepatic cholangiocarcinoma is a risk factor associated with hepatolithiasis. In this study, we retrospectively analyzed cases of patients with distal cholangiocarcinoma diagnosed after the removal of choledocholithiasis.

## 2. Patients and Methods

### 2.1. Patients

Cholangiocarcinoma is anatomically classified as intrahepatic or extrahepatic. Extrahepatic cholangiocarcinoma is classified as either perihilar or distal tumors according to the distance from the cystic ducts. This was a retrospective study of 46 patients with distal cholangiocarcinoma who underwent surgical treatment between April 2007 and December 2011. We assigned 9 cases of cholangiocarcinoma with choledocholithiasis to Group A; these cases accounted for 2.9% of all patients treated endoscopically for choledocholithiasis at our institution. As a control group, 37 patients with cholangiocarcinoma who did not undergo choledocholithiasis resection during the same period were assigned to Group B. Clinical features, endoscopic retrograde cholangiopancreatography (ERCP) findings, and histological diagnoses were analyzed retrospectively. Final pathological reports were reviewed to confirm the diagnosis of distal cholangiocarcinoma. The diameter of the distal bile duct and the morphology of the bile duct narrowing were also analyzed using the ERCP images.

### 2.2. Endoscopic Treatment

All patients with choledocholithiasis diagnosed by radiological visualization (ultrasonography (US), computed tomography (CT), magnetic resonance cholangiopancreatography) underwent endoscopic removal of bile duct stones. After stone removal, cholangiography was performed to confirm the complete removal of choledocholithiasis. Occasionally, owing to some limitations in the detection of small stones and sludge, they were not detected by cholangiography. Therefore, we performed intraductal US (IDUS) for all cases because of residual stones.

### 2.3. Surgical Procedure

Patients with distal tumors generally underwent pancreaticoduodenectomy with or without preservation of the pylorus. All patients underwent dissection of the regional lymph nodes, except for the para-aortic lymph nodes.

### 2.4. Data Collection

Preoperative demographic and clinical data and pathologic diagnosis data were collected prospectively.

### 2.5. Statistical Analysis

Continuous data were expressed as mean ± standard deviation (SD). The *χ* ^2^ test was used to compare qualitative parameters, and the Student *t-*test was used for quantitative parameters. *P* values of <0.05 were considered significant.

## 3. Results

### 3.1. Patient Characteristics

Three hundred and eleven patients who were diagnosed with choledocholithiasis between April 2007 and December 2011 underwent ERCP at our institution. Nine of them (2.9%) were diagnosed with distal cholangiocarcinoma by ERCP or IDUS after stone removal despite the tumors not being detected by radiological visualization. There were no statistical differences between the patient groups regarding age or gender. The only significant difference between Group A and Group B (*P* = 0.001) was in terms of abdominal pain at admission, and other clinical presentations were similar between both groups. All patients in Group A had gall bladder stones, compared with 7 patients (19%) in Group B (*P* < 0.01) (Table 1). The clinicopathological findings for Group A are summarized in Table 2.

### 3.2. Radiological Findings

The diagnostic imaging test results are shown in Table 3. In 7 patients in Group A, US detected no tumor in 3 patients (43%) and a dilatation of the bile duct in 4 patients (57%). In 34 patients in Group B, US detected no tumor in 7 patients (21%), a dilatation of the bile duct in 12 patients (35%), and a tumor in 15 patients (44%) (*P* = 0.083). Of the 9 patients in Group A, CT detected no tumor in 6 patients (67%) and a dilatation of the bile duct in 3 patients (33%). In 35 patients in Group B, CT detected no tumor in 6 patients (17%), a dilatation of the bile duct in 9 patients (26%), and a tumor in 20 patients (57%) (*P* = 0.003). Of the 9 patients in Group A, ERCP detected normality in 2 patients (22%) and abnormalities in 7 patients (78%). Of the 32 patients in Group B, ERCP detected normality in 4 patients (13%) and abnormalities in 28 patients (88%) (*P* = 0.597). IDUS detected a tumor in 8 patients in Group A, while in Group B, IDUS detected normality in 1 patient (3%) and tumors in 29 patients (97%) (*P* = 1.000).

### 3.3. Endoscopic Retrograde Cholangiopancreatography Findings

A significant difference was observed in the diameter of the common bile duct between the 2 groups (*P* = 0.043). The morphology of bile duct narrowing was classified as normal or mild irregularity, unilateral narrowing, or bilateral narrowing. The bile duct morphologies in the Group A patients were normal or mild irregularity in 3 patients (33%), unilateral narrowing in 5 patients (56%), and bilateral narrowing in 1 patient (11%). Of the Group B patients, 5 (14%) presented with normal or mild irregularity, 12 (32%) with unilateral narrowing, and 20 (54%) with bilateral narrowing (Table 4).

### 3.4. Histological Findings

Tumor size was not significantly different between the groups. The histological type of the distal cholangiocarcinoma in all (100%) Group A patients (9 patients in total) was well-differentiated adenocarcinoma. In Group B patients, the histological types of the distal cholangiocarcinoma were papillary adenocarcinomas in 3 patients (8%), well-differentiated adenocarcinoma in 20 patients (54%), moderately differentiated adenocarcinoma in 11 patients (30%), and poorly differentiated adenocarcinoma in 3 patients (8%). In Group A, the depth of invasion reached the mucosa in 5 patients (56%), the fibromuscular layer in 2 patients (22%), the subserous layer in 1 patient (11%), and the serosa in 1 patient (11%). In Group B, the depth of invasion reached the mucosa in 7 patients (19%), the fibromuscular layer in 5 patients (14%), the subserous layer in 9 patients (24%), the serosa in 7 patients (19%), and the serosal infiltration in 9 patients (24%). In 7 patients (78%) in Group A, the cholangiocarcinoma invaded the mucosa and the fibromuscular layer at an early stage. The cholangiocarcinoma invaded the subserous layer: invaded the serosa in 22% of the patients (2/9) and infiltrated the serosa in 68% of the patients (25/37) (*P* = 0.022). In Group A, lymph node classification was absent in 8 patients (89%) and present in 1 patient (11%). In Group B, the lymph node classification was absent in 24 patients (65%) and present in 13 patients (35%) (*P* = 0.234) (Table 5).

### 3.5. Prognoses

All patients in Group A were alive. In Group B, the median survival time was 46 months. Survival time was not significantly different between the 2 groups (*P* = 0.126). Furthermore, survival time was not influenced by the existence of choledocholithiasis.

## 4. Discussion

Because most patients with cholangiocarcinoma tend to invade the surrounding vessels and nerves, they are unresectable at the time of diagnosis, and consequently patient survival is poor. Early detection and diagnosis are essential for improving long-term survival because the 5-year survival rates of patients with distal cholangiocarcinoma have been reported to be 23% [2]. Ekbom et al. [3] reported that gall bladder stones are a probable risk factor for extrahepatic bile duct cancer. However, choledocholithiasis has not been reported as a cause of extrahepatic cholangiocarcinoma to date. Reports of distal cholangiocarcinoma associated with choledocholithiasis are very rare, and the causal relationship remains to be established. Because we observed early distal cholangiocarcinoma after stone removal, we investigated the probable role of choledocholithiasis as a risk factor. Kimura et al. [4] described the relationship between extrahepatic bile duct carcinoma and stones in autopsy cases. Extrahepatic bile duct carcinomas were present in 7 of 143 patients (4.9%) with stones, which was significantly higher than the rate in the patients without stones (26 of 4339; 0.6%) (*P* < 0.01). Nishimura et al. [5] also reported the relationship between distal cholangiocarcinoma and cholidocholithiasis. The incidence of intrahepatic cholangiocarcinoma associated with hepatolithiasis as a risk factor has been reported to be 2.4–5.4% [6–8]. Chronic inflammation, biliary infection, and cholestasis due to hepatolithiasis lead to cholangiocarcinoma as a result of chronic inflammation in the biliary epithelium. Furthermore, Terada and Nakanuma reported that carcinogenesis in biliary epithelia in livers with stones was a multistep process involving hyperplasia, dysplasia, and adenocarcinoma [9]. We considered the possibility that stones may also be associated with distal cholangiocarcinoma as well as being a risk factor for intrahepatic cholangiocarcinoma. In the present study, in 1 case of stone impaction, we found that the tumor was located proximal to the stone. Because all the other tumors were distal to the stones, persistent chronic stimulation by stones rather than cholestasis and infection may lead to carcinogenesis in the biliary epithelium.

The recurrence rate of choledocholithiasis after stone removal has been reported to be 24% [10–12]. Therefore, it is possible that a cholangiogram obtained immediately after stone removal underestimates residual stones owing to numerous air bubbles entering the bile duct from the sphincterotomy. IDUS after stone removal showed residual stones in 33–40% of cases [13, 14], although cholangiography did not detect them. Therefore, IDUS after stone removal is useful because the sensitivity of IDUS for detecting choledocholithiasis is also very high [15]. In addition, a prospective study for the utility of IDUS has been reported [16]. Additional IDUS to confirm complete stone clearance decreases the early recurrence rate of choledocholithiasis. For example, the recurrence rate was 13.2% in the non-IDUS group and 3.4% in the IDUS group (*P* < 0.05), and multivariate analysis identified additional IDUS as an independent risk factor for the recurrence of bile duct stones.

Three hundred and eleven consecutive patients who underwent ERCP for choledocholithiasis between April 2005 and December 2011 were included in the study. All patients underwent IDUS after stone removal. Fortunately, IDUS detected biliary strictures in 2.9% of cases (9/311) that were pathologically diagnosed as cholangiocarcinoma. In each case, IDUS initially detected a cholangiocarcinoma in the absence of a mass on CT or magnetic resonance imaging. Because the early distal cholangiocarcinomas in 39% of cases (7/18) in our institution were associated with choledocholithiasis, we suggest that choledocholithiasis shows an etiologic association with cholangiocarcinoma. IDUS is very useful for evaluating not only residual stones but also biliary strictures [17, 18]. Because IDUS can be performed easily and safely over a guidewire, we performed routine additional IDUS with ERCP in all cases. In this study, ERCP and IDUS in Group A detected biliary strictures without tumor lesions on US or CT in 7 of 9 cases and all cases, respectively. Because additional IDUS may underestimate the coexistence of cholangiocarcinoma after stone removal, it should be performed carefully.

In another study, histological grading indicated that the cholangiocarcinoma in a group of hepatolithiasis patients exhibited a significantly higher percentage of well-differentiated tumors [19]. Because all cases of cholangiocarcinoma associated with choledocholithiasis were pathologically diagnosed as well differentiated, the result of this study is similar to that of our study. Chronic stimulation of biliary epithelium by stones may be associated with well-differentiated cholangiocarcinoma. It has been hypothesized that carcinogenesis in the biliary epithelium in livers with hepatolithiasis is a multistep process that follows a hyperplasia-dysplasia-carcinoma sequence [9, 20, 21]. In a study on the carcinogenic process in patients with cholangiocarcinoma arising from pancreaticobiliary malfunction, it was hypothesized that carcinogenesis is involved in chronic inflammation in the biliary epithelium and genetic abnormalities in *K-ras*, *p53*, *MUC1*, and *COX2* occurred after chronic inflammation [22, 23].

In conclusion, IDUS after stone removal may potentially help in the detection of unexpected tumors. Therefore, we believe that IDUS after stone removal will lead to improve outcome and prognosis. We also hope that this study will assist in the understanding of both distal cholangiocarcinoma associated with choledocholithiasis and the molecular mechanisms underlying choledocholithiasis-related distal cholangiocarcinoma, for which only limited data are available. However, further studies with a higher number of cases are required to support the findings presented here.

## Figures and Tables

**Table 1:** Patient characteristics.

	Group A	Group B	*P* value
	*n* = 9	*n* = 37	
Age	68.8 ± 9.2	69.6 ± 9.7	0.815
Gender			
Male : female	4 : 5	27 : 10	0.127
Symptom			
Jaundice	6	29	0.460
Abdominal pain	7	8	0.001
Fever	2	5	0.609
Anorexia	0	10	0.172
Asymptomatic	2	5	0.609
Associated disease			
GS	9	7*	<0.01
Cholangitis	2	4	0.581

*following cholecystectomy in one case.

**Table 2:** Summary of the clinicopathological findings of Group A.

Case	Age	Gender	Clinical presentation	Cholangiographic finding	Differentiation	Depth	Prognosis (months)	
1	60	Male	Jaundice, abdominal pain, fever	Unilateral narrowing	well	m	11	Alive
2	74	Male	Asymptomatic	Unilateral narrowing	well	m	5	Alive
3	79	Female	Asymptomatic	Bilateral narrowing	well	m	5	Alive
4	62	Female	Abdominal pain	Normal	well	m	3	Alive
5	61	Female	Jaundice, abdominal pain	Normal	well	m	3	Alive
6	59	Male	Jaundice, abdominal pain	Unilateral narrowing	well	fm	45	Alive
7	84	Female	Jaundice, abdominal pain	Unilateral narrowing	well	fm	2	Alive
8	74	Male	Jaundice, abdominal pain, fever	Unilateral narrowing	well	ss	60	Alive
9	66	Female	Jaundice, abdominal pain	Mild irregularity	well	se	81	Alive

m: mucosa, fm: fibromuscular layer, ss: subserous layer, se: serosa.

**Table 3:** Radiologic findings.

	Group A	Group B	*P* value
	*n* = 9	*n* = 37	
US			
Normal	3	7	0.083
Dilatation	4	12	
Tumor	0	15	
CT			
Normal	6	6	0.003
Dilatation	3	9	
Tumor	0	20	
ERCP			
Normality	2	4	0.597
Abnormality	7	28	
IDUS			
Normality	0	1	1.000
Tumor	8	29	

**Table 4:** Endoscopic retrograde cholangiopancreatography findings.

	Group A	Group B	*P* value
	*n* = 9	*n* = 37	
Diameter of the common bile duct	9.7 ± 2.1	13.0 ± 4.7	0.043
Morphology of the bile duct			0.061
Normal or mild irregularity	3	5	
Unilateral narrowing	5	12	
Bilateral narrowing	1	20	

**Table 5:** Histological findings.

	Group A	Group B	*P* value
	*n* = 9	*n* = 37	
Tumor size	15.3 ± 9.7	24.6 ± 14.1	0.081
Depth of invasion			
Mucosa	5	7	1.130
Fibromuscular layer	2	5	
Subserous layer	1	9	
Serosa	1	7	
Serosa infiltrating		9	
Subserous layer—deeper	2 (22%)	25 (68%)	0.022
Differentiation			
Papillary	0	3	0.087
Well	9	20	
Moderately	0	11	
Poorly	0	3	
Lymph node involvement	1	13	0.240
